# Supplementary material for: Paired DNA and RNA sequencing uncovers common and rare genetic variants regulating gene expression in the human retina
Source: medRxiv. 2025 Apr 26:2025.04.25.25326445. Preprint. [Version 1] doi: 10.1101/2025.04.25.25326445 (PMC12045431; doi:10.1101/2025.04.25.25326445)
Supplement: Supplement 1 [file NIHPP2025.04.25.25326445v1-supplement-1.pdf]

medRxiv preprint doi: https://doi.org/10.1101/2025.04.25.25326445; this version posted April 26, 2025. The copyright holder for this preprint (which was not certified by peer review) is the author/funder, who has granted medRxiv a license to display the preprint in perpetuity.
It is made available under a CC-BY 4.0 International license .

Supplementary Figures

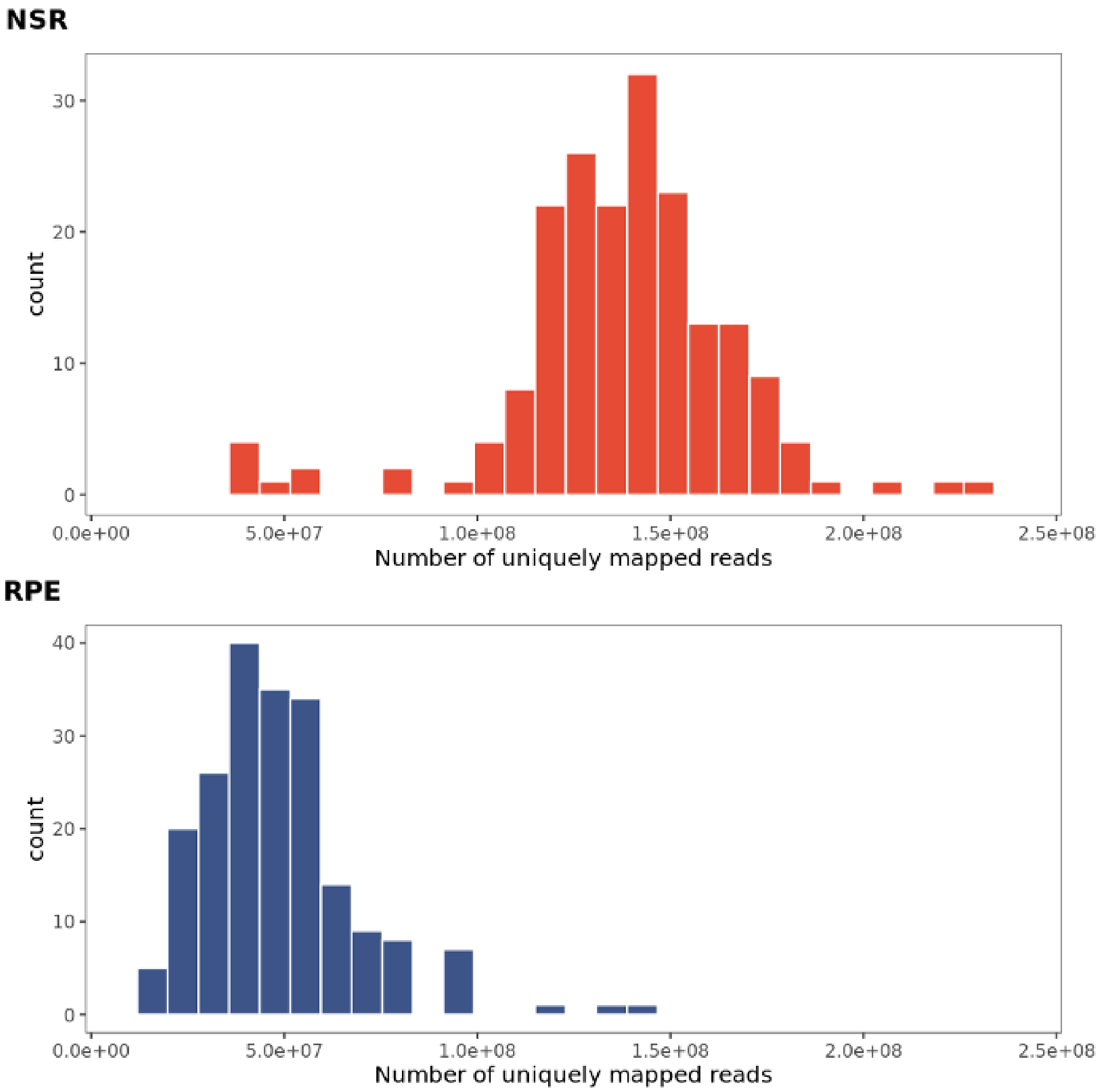

Supplementary figure 1 Number of uniquely mapped reads in NSR (n = 183) and RPE (n = 176) samples from the METR-GT cohort

medRxiv preprint doi: https://doi.org/10.1101/2025.04.25.25326445; this version posted April 26, 2025. The copyright holder for this preprint (which was not certified by peer review) is the author/funder, who has granted medRxiv a license to display the preprint in perpetuity.
It is made available under a CC-BY 4.0 International license .

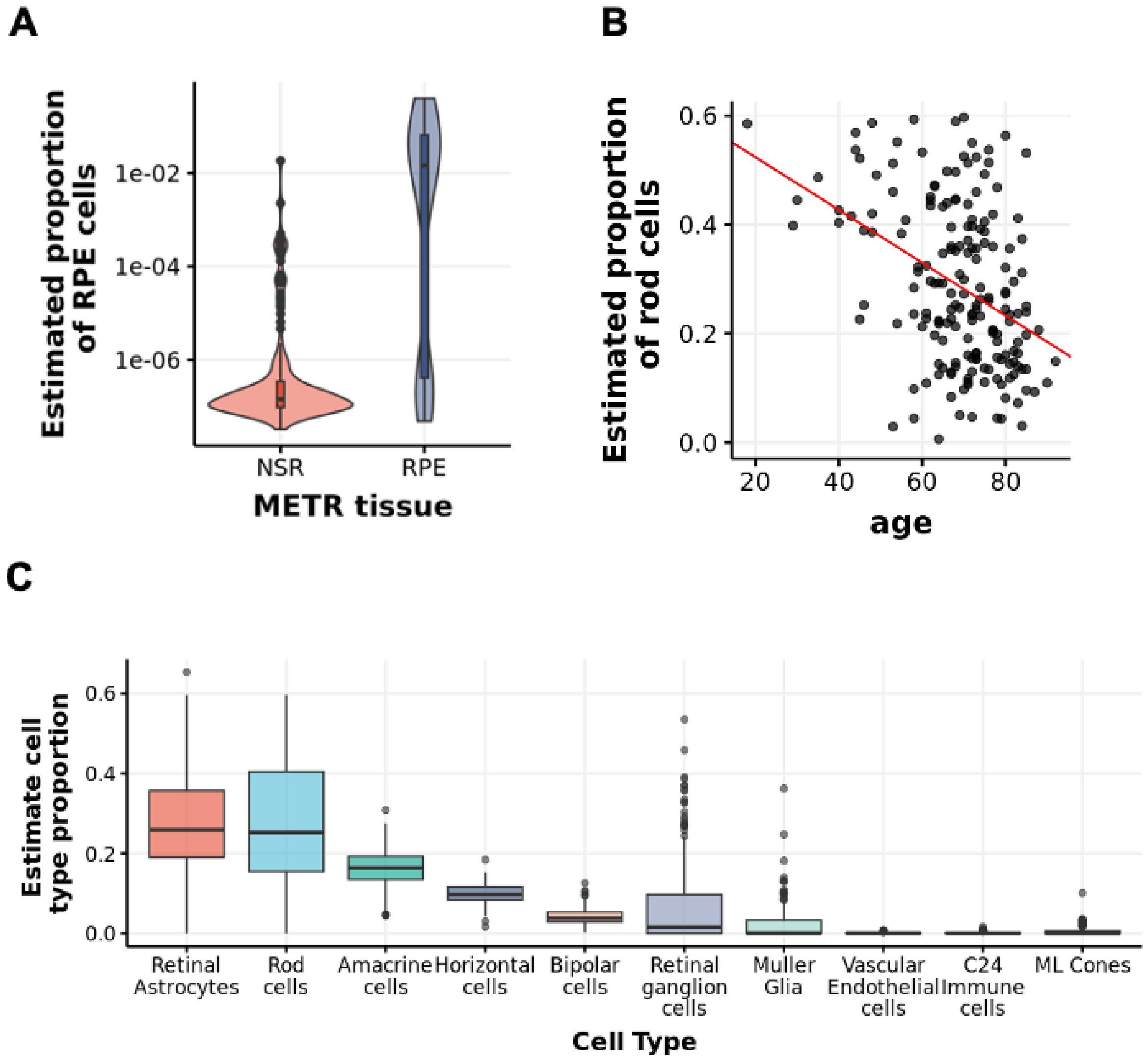

Supplementary figure 2 Cell type deconvolution of METR bulk RNASeq samples *A. The estimated proportion of RPE cells in METR-RPE samples is higher than in the METR-NSR cohort. B. The estimated proportion of rod cells decreases in samples from older donors. C. The estimated proportion of individual cell types across the METR-NSR cohort.*

375

376

medRxiv preprint doi: https://doi.org/10.1101/2025.04.25.25326445; this version posted April 26, 2025. The copyright holder for this preprint (which was not certified by peer review) is the author/funder, who has granted medRxiv a license to display the preprint in perpetuity.
It is made available under a CC-BY 4.0 International license .

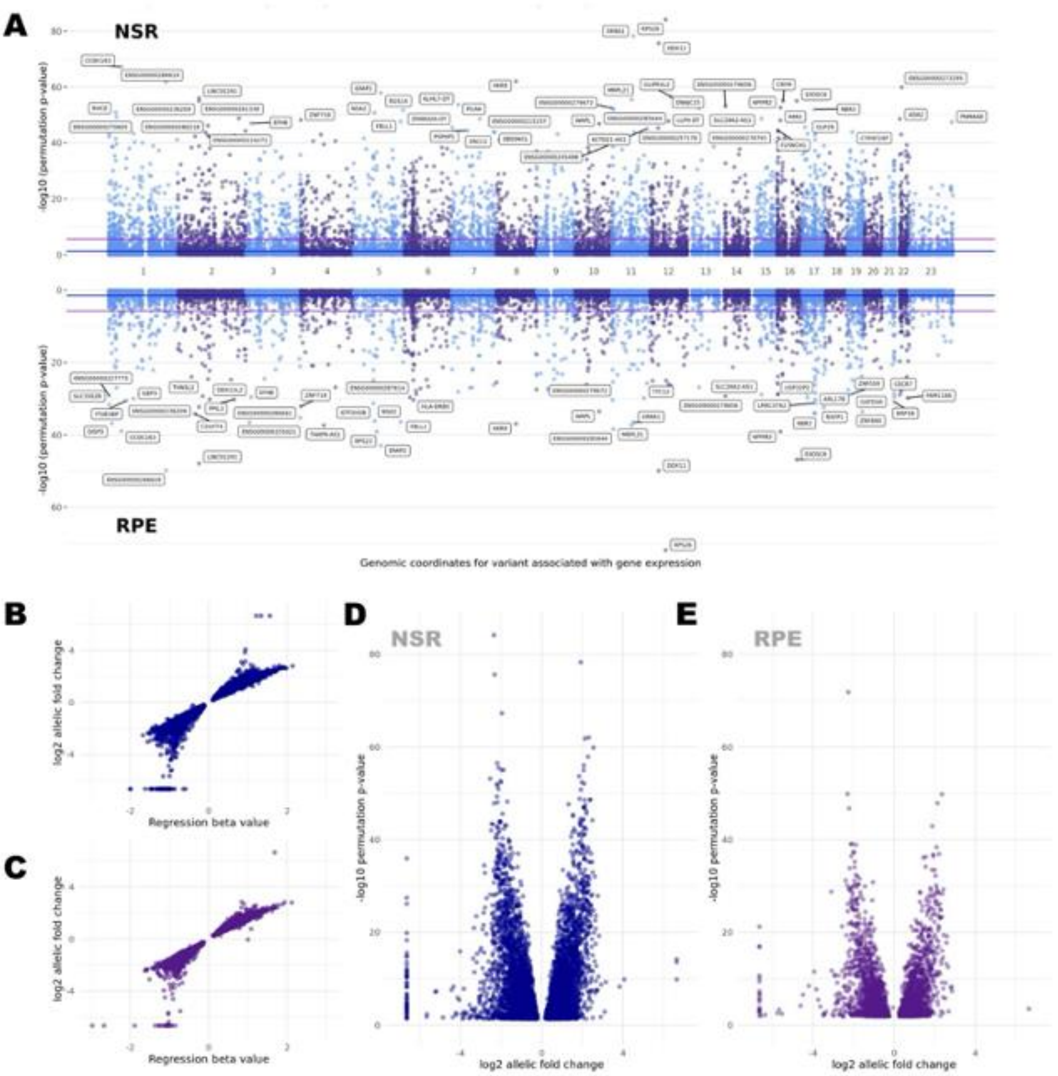

Supplementary figure 3 Cis-eQTL mapping in NSR and RPE based on a permutation model to identify the top eQTL candidate per gene. A) *Manhattan plot of the top cis-eQTLs per gene in the NSR (top) and the RPE (bottom). The blue line indicates the False Discovery Rate (FDR) threshold of 5% used to identify genes with a significant eQTL (eGenes). In the NSR we identified 8,609 eGenes and 3,229 in the RPE. The purple line indicates the more conservative Bonferroni corrected p-value threshold 0.05).* ***B)*** *Relationship between the regression beta values computed for each of the top eQTLs in the NSR and the log2 allelic fold change. The magnitude of the beta value has no direct biological interpretation as it is computed based on transformed expression values.* ***C)*** *Relationship between the regression beta values computed for each of the top eQTLs in the RPE and the log2 allelic fold change.* ***D)*** *Volcano plot of the log2 allelic fold change for each of the top eQTLs in NSR* ***E)*** *Volcano plot of the log2 allelic fold change for each of the top eQTLs in RPE*

377

378

medRxiv preprint doi: https://doi.org/10.1101/2025.04.25.25326445; this version posted April 26, 2025. The copyright holder for this preprint (which was not certified by peer review) is the author/funder, who has granted medRxiv a license to display the preprint in perpetuity.
It is made available under a CC-BY 4.0 International license .

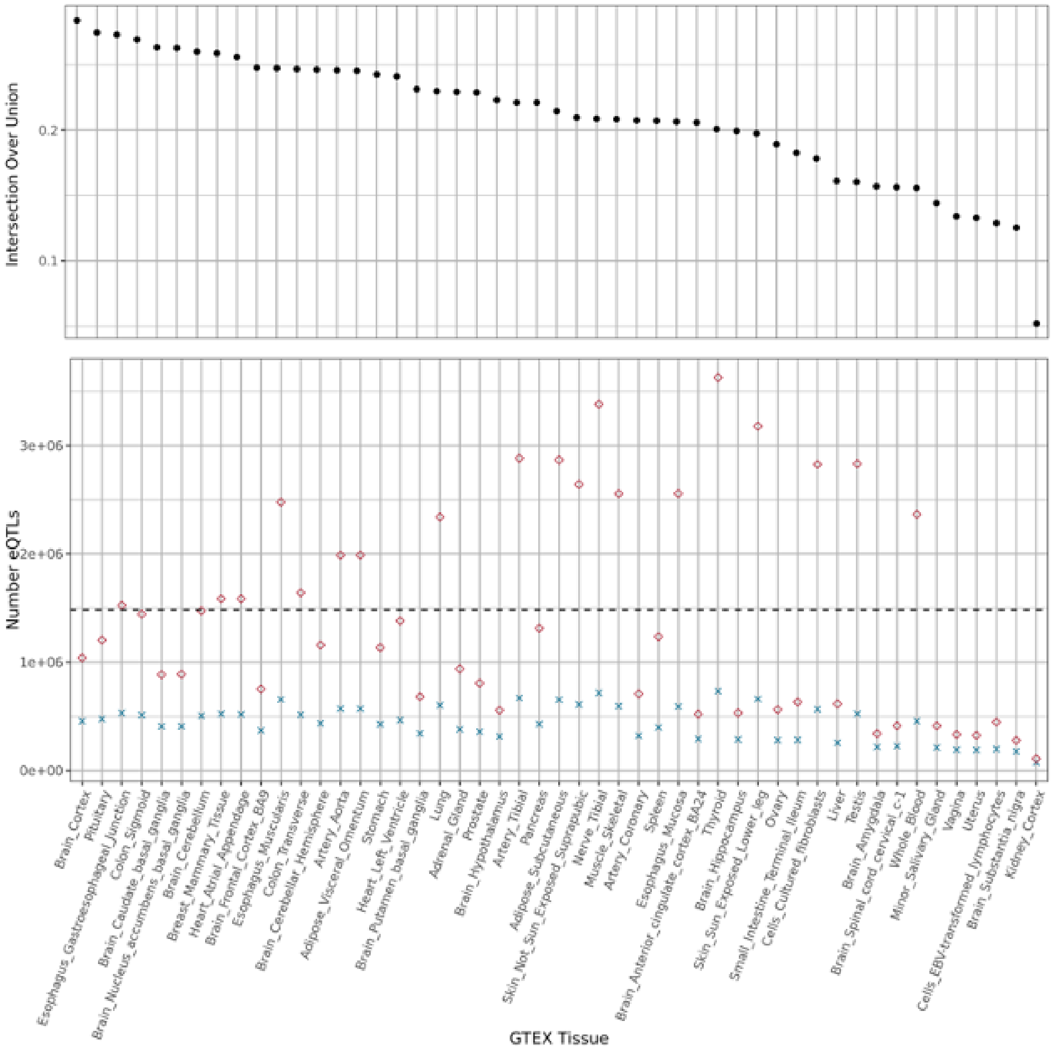

Supplementary figure 4 *Intersection of the eQTLs identified in NSR and/or RPE with each GTEx study (v8). The bottom plot indicates the number of shared eQTLs in NSR/RPE and each tissue (cross) and the overall number of eQTLs identified in each GTEx study (circle). The top plot indicates relative proportion of eQTLs identified in each tissue and our study (intersection over union).*

medRxiv preprint doi: https://doi.org/10.1101/2025.04.25.25326445; this version posted April 26, 2025. The copyright holder for this preprint (which was not certified by peer review) is the author/funder, who has granted medRxiv a license to display the preprint in perpetuity.
It is made available under a CC-BY 4.0 International license .

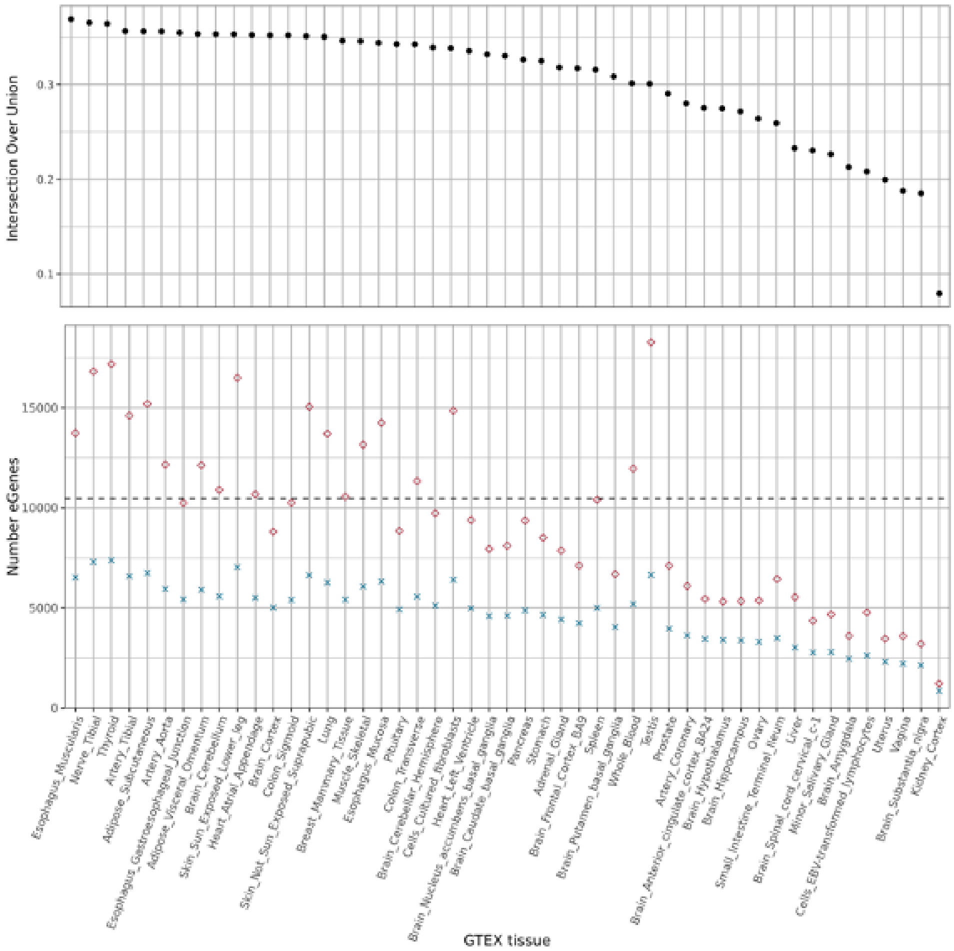

Supplementary figure 5 Intersection between eGenes in each GTEX tissue and the METR-eGenes identified in the NSR and/or RPE.

medRxiv preprint doi: https://doi.org/10.1101/2025.04.25.25326445; this version posted April 26, 2025. The copyright holder for this preprint (which was not certified by peer review) is the author/funder, who has granted medRxiv a license to display the preprint in perpetuity.
It is made available under a CC-BY 4.0 International license .

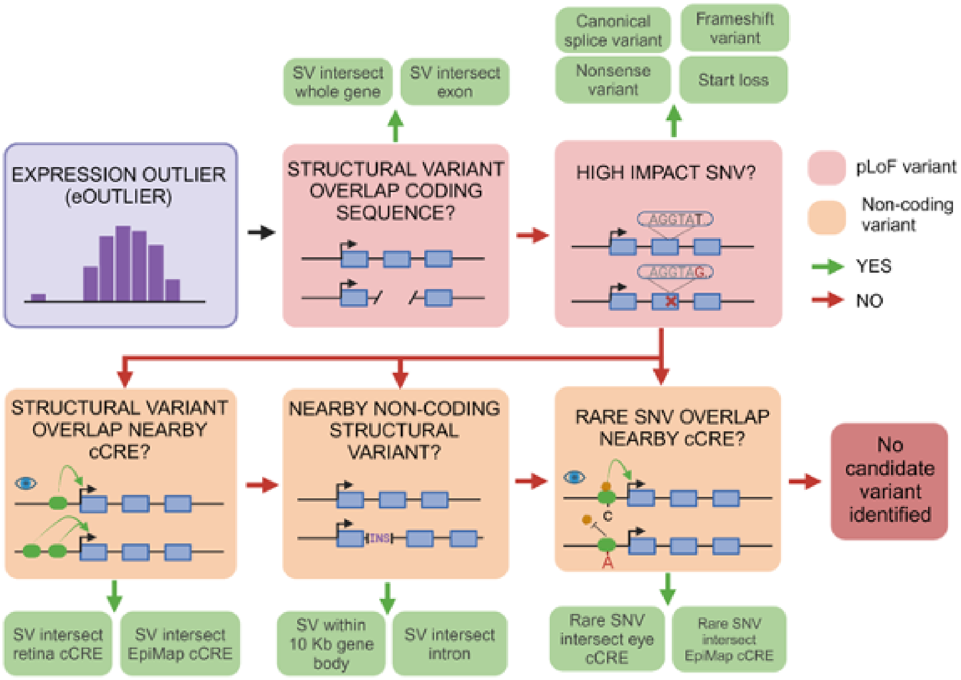

Supplementary figure 6 Overview of the hierarchical workflow to identify candidate variants driving outlier expression

383

384
